# Abnormal Road Surface Recognition Based on Smartphone Acceleration Sensor

**DOI:** 10.3390/s20020451

**Published:** 2020-01-13

**Authors:** Ronghua Du, Gang Qiu, Kai Gao, Lin Hu, Li Liu

**Affiliations:** 1 College of Automotive and Mechanical Engineering, Changsha University of Science & Technology, Changsha 410114, China; csdrh@csust.edu.cn (R.D.); qiugang.px@stu.csust.edu.cn (G.Q.); hulin@csust.edu.cn (L.H.); lukeliuli@csust.edu.cn (L.L.); 2 Hunan Key Laboratory of Smart Roadway and Cooperative Vehicle-Infrastructure Systems, Changsha University of Science & Technology, Changsha 410114, China

**Keywords:** road surface recognition, Gaussian background model, abnormal road surface, acceleration sensor

## Abstract

In order to identify the abnormal road surface condition efficiently and at low cost, a road surface condition recognition method is proposed based on the vibration acceleration generated by a smartphone when the vehicle passes through the abnormal road surface. The improved Gaussian background model is used to extract the features of the abnormal pavement, and the k-nearest neighbor (kNN) algorithm is used to distinguish the abnormal pavement types, including pothole and bump. Comparing with the existing works, the influence of vehicles with different suspension characteristics on the detection threshold is studied in this paper, and an adaptive adjustment mechanism based on vehicle speed is proposed. After comparing the field investigation results with the algorithm recognition results, the accuracy of the proposed algorithm is rigorously evaluated. The test results show that the vehicle vibration acceleration contains the road surface condition information, which can be used to identify the abnormal road conditions. The test result shows that the accuracy of the recognition of the road surface pothole is 96.03%, and the accuracy of the road surface bump is 94.12%. The proposed road surface recognition method can be utilized to replace the special patrol vehicle for timely and low-cost road maintenance.

## 1. Introduction

During the operation of the road, the road surface will inevitably suffer from some defects or damage due to the crushing, impact, and weather changes of the passing vehicles. These defects and damage are often referred to as abnormal road conditions [1]. Abnormal road conditions have a negative impact on vehicle speed, fuel consumption, mechanical wear, ride comfort, and even safety. The traditional abnormal road condition information collection mainly relies on the manual site survey and special patrol vehicle, which is inefficient and high in cost. According to the statistics of the Ministry of Transport of China, the maintenance expenditure of the national toll road in 2017 has reached 53.39 billion yuan.

In order to save money and time costs, many experts and scholars have studied abnormal road surface identification methods [2,3]. There are three main methods for identifying abnormal roads: Visual [4], three-dimensional reconstruction [5,6], and dynamic vehicle response. The method of road surface recognition based on visual or three-dimensional reconstruction has high-performance requirements and high cost, which is not conducive to comprehensive promotion and use. The test scope is also very limited. The method based on the smartphone acceleration sensor to identify the abnormal road surface has certain advantages [2].

In early research, accelerometers are used for pavement recognition. De Zoysa [7] proposed to deploy a small number of mobile sensors in the public transportation system to detect road conditions, but the system exhibited low detection efficiency and a false-positive rate. Chen [8] proposed a crowdsourcing based road surface monitoring system. By adding acceleration sensors and GPS modules to the vehicle to obtain the acceleration, velocity, and position information of the vehicle, the Gaussian background model is used to identify the abnormal road surface. Based on the research of Chen, Harikrishnan [9] improved the Gaussian background model and proposed an abnormal road surface recognition method that can adapt to different vehicle speeds. This method could classify the speed bump and road surface bumps according to the X-Z axis acceleration ratio.

In order to improve the accuracy of road surface recognition, Wang [10] proposed a method by fusing the feature data from an acceleration sensor and camera to identify the abnormal road type. Celaya [11] installed sensors at the front of the vehicle to obtain the vehicle vibration response when the vehicle passed the speed bump and used the multivariate genetic algorithm to detect the road surface anomaly. This method can realize the recognition of abnormal road surfaces with a low false alarm rate, but the calculation is complicated, and a large number of statistical features such as mean, variance, peak, and standard deviation are needed for machine learning. With the rapid development of mobile intelligent terminal technology, smartphones equipped with sensors such as accelerometers and global positioning navigation systems can be used to detect abnormal road surfaces [12]. Cong [13] used the probabilistic statistical method and wavelet analysis method to establish an identification model of abnormal data and uses median filtering and wavelet filtering to process the data so that the data detected by the smartphone can truly reflect the vibration of the vehicle. Yi [14] proposed a smartphone detection vehicle to monitor the road surface, using an anomaly indexing algorithm to detect the speed bump, the pit, and the manhole cover. However, in the subsequent experiments, it was found that the proposed algorithm can only identify the speed bump, and the identification of other abnormal road conditions is not favorable. Mukherjee [15] established a quarter-vehicle model and a half-vehicle model and studied the acceleration response of the vehicle when crossing the deceleration belt. They developed a mathematical statistics method to identify the deceleration belt. Zhao [16] proposed a feature extraction method combining time-domain parameter characteristics and wavelet packet energy characteristics based on vehicle suspension vibration response and used a probabilistic neural network to classify road surface.

In summary, the research on the road surface condition identification method has achieved certain results. Recent studies have proven that smartphone accelerometers can effectively capture vehicle vibrations caused by abnormal road surfaces. By analyzing the signals from these mobile sensors, we have the potential to identify road anomalies. In this study, a Gaussian background model is used to identify abnormal roads, and an adaptive adjustment mechanism based on vehicle speed is proposed to improve the recognition accuracy. The parameters of the Gaussian background model are optimized by using fuzzy logic inference machines, making the method suitable for different types of vehicles, and using the kNN algorithm to classify abnormal roads.

## 2. Road Information Sharing System

Obtaining abnormal road information in advance can effectively prevent traffic accidents, but due to road geometry, weather, lighting conditions, etc., the driver may not be able to notice the abnormal road ahead. The information-sharing technology is used to construct an abnormal road sharing system, especially encouraged by the development of the vehicle network and intelligent transportation system [17,18,19,20,21]. The road information sharing system is designed to promptly warn the driver when the driver approaches an abnormal road at a dangerous speed. In addition, the relevant information of the abnormal road surface is sent to the municipal road maintenance unit, so that the road maintenance personnel can timely get the road damage status and repair it to ensure the safety and comfort of travel [22,23,24,25].

**Figure 1:**
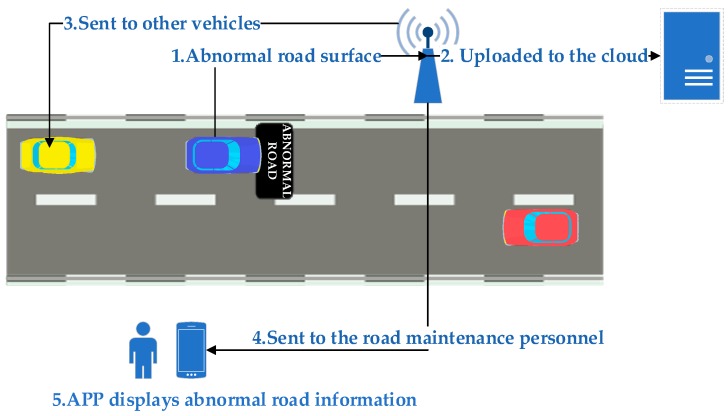
Road information sharing system architecture diagram.

In order to monitor the road surface conditions in realtime, it is necessary to find an efficient and reliable communication technology to transmit abnormal road information. This paper uses existing cellular network technology to collect abnormal road surface data. Figure 1 shows an architectural diagram of a road information sharing system. A smartphone with an acceleration sensor and a GPS module is fixed on the vehicle to obtain the latitude and longitude coordinates of the vehicle, the traveling speed, and the vibration acceleration data of the vehicle body. When the vehicle passes the abnormal road surface, the smartphone will upload the location and type of the abnormal road surface to the cloud. The abnormal road information is sent to the road maintenance personnel. When other vehicles approach the abnormal road surface, the cloud will issue an abnormal road surface reminder to ensure that the vehicle can pass the area safely and smoothly.

## 3. Data Processing

There are many sources of vibration in a vehicle. Different vibration sources have certain differences in frequency domain characteristics. In this study, a quarter of the vehicle model and the abnormal road surface model are established to analyze the excitation effect of the abnormal road on the overall vehicle system. In order to obtain the vibration frequency range of the vehicle body, a frequency spectrum analysis of the vibration acceleration of the vehicle body is implemented, and a filter based on this analysis result is designed.

### 3.1. Vehicle Dynamics Analysis

**Figure 2:**
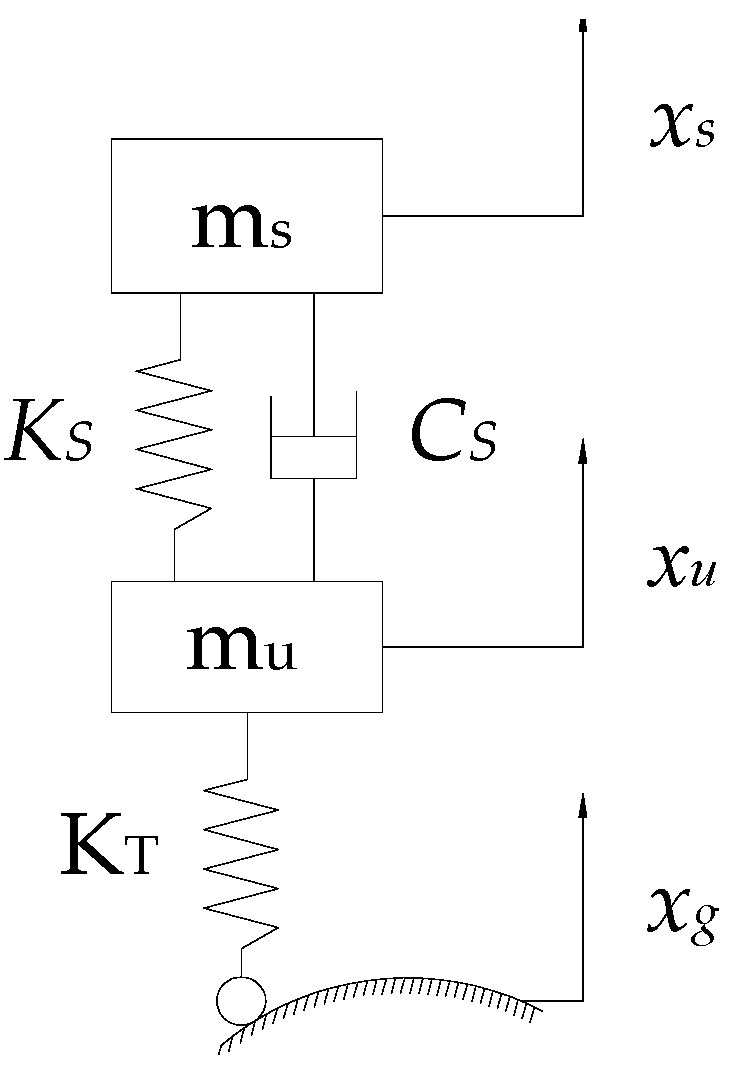
The quarter vehicle model.

In order to study the interaction between the vehicle and the road, a quarter-vehicle model is utilized to analyze the vibration of the vehicle in the vertical direction. The simplified vehicle dynamics model is shown in Figure 2.

m_u_ is the unsprung mass. m_s_ is the sprung mass. *K_S_* is the stiffness of the spring. *C_S_* is the damping coefficient of the shock absorber. K_T_ is the stiffness of the tire. *x_s_* is the vertical displacement of the vehicle body. *x_u_* is the vertical displacement of the wheel. *x_g_* is the road surface excitation. The differential equation of motion of the vehicle is described as Equation (1).

(1) $$\left\{ \begin{array}{l} m_{s}{\ddot{x}}_{s}+C_{S}({\dot{x}}_{s}-{\dot{x}}_{u})+K_{S}(x_{s}-x_{u})=0\\ m_{u}{\ddot{x}}_{u}-C_{S}({\dot{x}}_{s}-{\dot{x}}_{u})-K_{S}(x_{s}-x_{u})+K_{T}(x_{u}-x_{g})=0 \end{array}\right.$$

**Figure 3:**
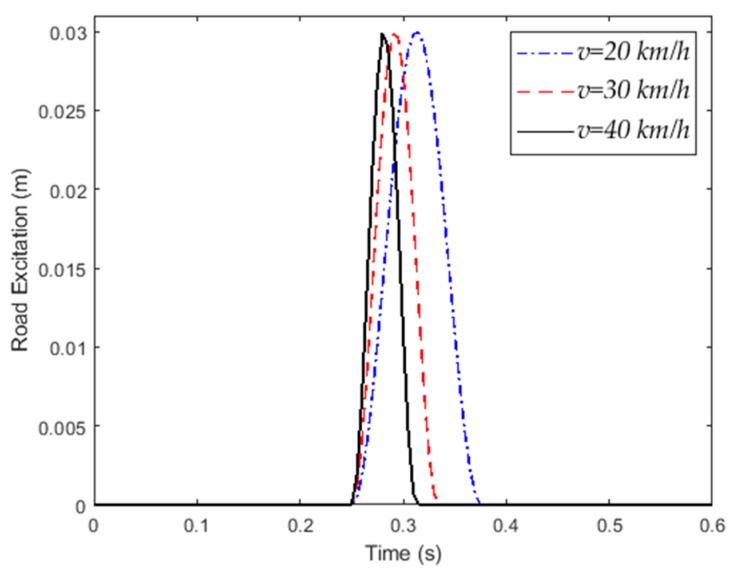
Abnormal road surface excitation at different speeds.

This paper analyzes the dynamic response of the vehicle when the vehicle passes through the road surface at different speeds [26]. An abnormal pavement model is shown in Figure 3. The length of the abnormal road surface is L, and the height is h. *v* is the speed of the vehicle. t_1_ and t_2_ are the starting moment and the ending moment of the road surface excitation, respectively. The mathematical model is described as Equation (2) [27].

(2) $$x_{g}(t)=\left\{ \begin{array}{l} 0.5h\left(1-\cos\left(\frac{2\pi v}{L}t\right)\right),t_{1}\leq t\leq t_{2}\\ 0,t_{1}<t\;\mathrm{or}\;t>t_{2} \end{array}\right.$$

**Figure 4:**
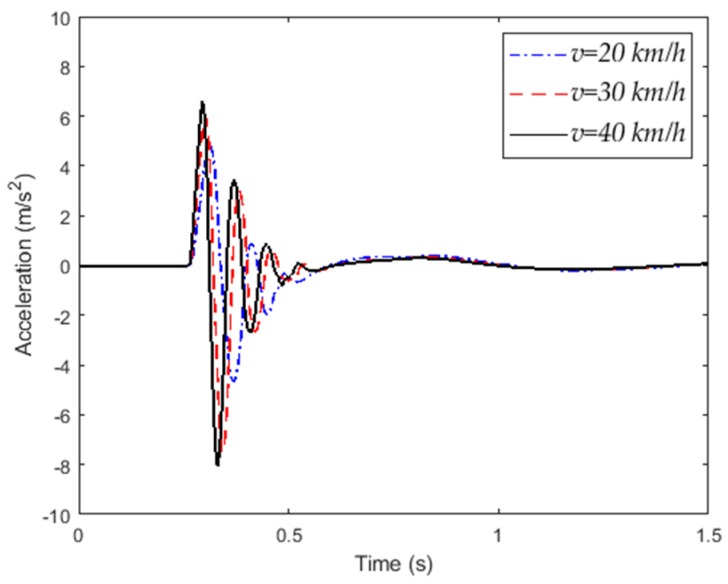
Vertical acceleration response of vehicle.

**Figure 5:**
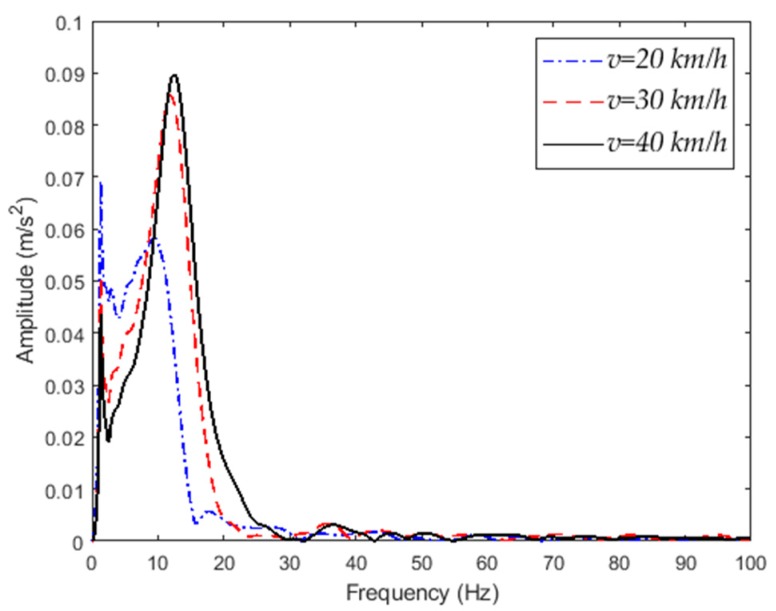
Vertical acceleration signal spectrum.

When the vehicle passes through an abnormal road at different speeds, the vertical acceleration response of the vehicle body is shown in Figure 4. The speed of the vehicle has a significant effect on the vertical acceleration of the vehicle. The frequency–domain analysis of the acceleration signal is performed to obtain a spectrogram of the vehicle vibration acceleration signal, as shown in Figure 5. As the vehicle speed increases, the frequency of body vibration increases, but its main component is still in the low-frequency range (30 Hz).

### 3.2. Butterworth Filter

When the data is collected, the position of the smartphone is not flat, and the slope of the test road surface may interfere with the recognition of the abnormal road surface. Therefore, the data needs to be filtered before using to recognize the abnormal road surface. According to the simulation data analysis results, the vibration caused by the abnormal road surface excitation is mainly distributed in the low-frequency range.

**Figure 6:**
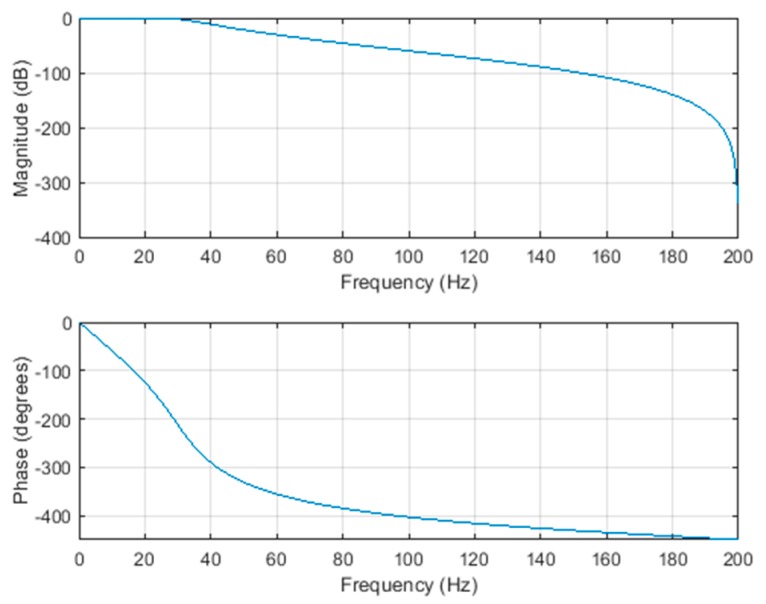
The frequency response of the Butterworth filter.

In this paper, the Butterworth filter is used to filter out the through component and the high-frequency noise with the frequency greater than 30 Hz. There are several reasons for choosing the Butterworth filter. Firstly, the Butterworth filter does not generate a ripple in the passband. Secondly, it has been successfully implemented in many commercial tools. Figure 6 shows the frequency response of the fifth-order Butterworth’s numerical low-pass filters with a cut-off frequency of 30 Hz.

In addition, the filter delay has a negative impact on the positioning accuracy of abnormal roads surface. The filter delay will shift the positioning of the abnormal road surface towards the vehicle driving direction for a distance. However, because the filtering delay is very small (about 0.1 s), the positioning error caused by the filtering delay is not large, and the positioning accuracy requirements for abnormal road recognition are not high. Therefore, the Butterworth filter can be applied to the recognition of abnormal roads.

## 4. Abnormal Road Surface Recognition

### 4.1. Overview of Abnormal Road Surface Recognition Algorithm

**Figure 7:**
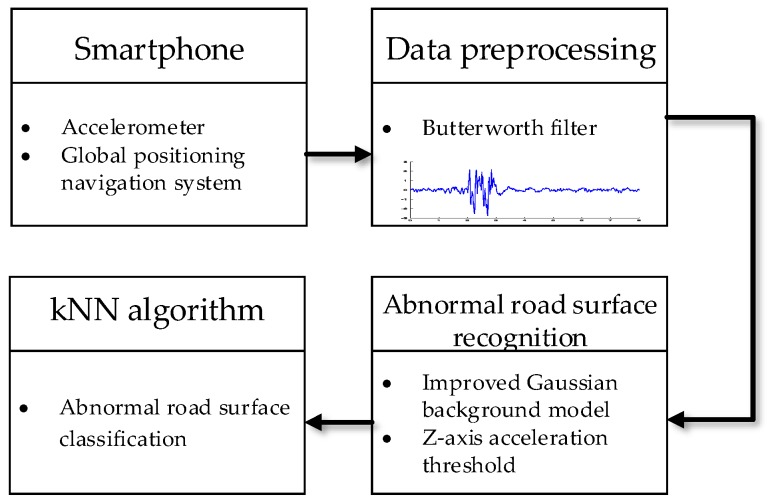
Road surface recognition algorithm framework.

The framework of the abnormal road recognition algorithm is shown in Figure 7. In order to collect vehicle speed, acceleration, and position information, the smartphone’s built-in accelerometer and global positioning navigation system are used. First, the raw data is preprocessed using a Butterworth filter. Secondly, the Gaussian background model is improved by using fuzzy logic control. The improved Gaussian model is combined with the acceleration threshold condition to extract the characteristic acceleration value caused by the abnormal pavement. Finally, the kNN algorithm is used to classify the abnormal road surface.

### 4.2. Gaussian Background Model

When the vehicle is traveling on a flat road surface, the vibration acceleration in the vertical direction of the vehicle is Gaussian-distributed. In order to verify the conclusion, we used both Kolmogorov–Smirnov and Lilliefors test methods to test whether the acceleration generated by the vehicle on a flat road conforms to the Gaussian distribution. The results show that the assumption is valid. By measuring the vibration acceleration of the vehicle, the vehicle’s vertical acceleration will be abrupt when the vehicle passes through an abnormal road surface compared to traveling on a flat road [28]. The Gaussian model is described as Equation (3).

(3) $$\eta \left(z|\mu ,\sigma^{2}\right)=\frac{1}{\sigma \sqrt{2\pi }}e^{\left(\frac{-{\left(z-\mu \right)}^{2}}{2\sigma^{2}}\right)}$$

*μ* is a mathematical expectation. *σ* is a standard deviation, and *z* is a body vibration acceleration value. If the vehicle passes over an abnormal road, the body acceleration at this time will no longer conform to the Gaussian distribution. Therefore, if *z* is the vertical acceleration of the vehicle caused by abnormal road excitation, the absolute value of the difference between *z* and *μ* is greater than the product of the threshold T_G_ and the standard deviation *σ*. The equation can be described as Equation (4).

(4) $$\left|\left(z-\mu \right)\right|>T_{G}*\sigma$$

If Equation (4) is not satisfied, the vehicle is considered to be traveling on a flat road. At this time, the background of the Gaussian model will change, and the model parameters will be updated. The Gaussian model parameter update equation is described as Equation (5).

(5) $$\left\{ \begin{array}{l} \mu_{t+1}=\left(1-\alpha \right)\mu_{t}+\alpha \left(z-\mu_{t}\right)\\ \sigma_{t+1}^{2}=\left(1-\alpha \right)\sigma_{t}^{2}+\alpha {\left(z-\mu_{t}\right)}^{2} \end{array}\right.$$

where α is the learning rate, and the size of the learning rate indicates the speed of the update. *μ_t_*_+1_ and *σ_t+_*_1_ are the updated mean and standard deviation.

### 4.3. Improved Gaussian Background Model

The vibration acceleration of the vehicle is affected by the traveling speed. When the vehicle passes over abnormal roads at a different speed, the amplitude of the vibration acceleration is different. In order to avoid false alarms at high speed and false negatives at low speed, the Gaussian model described as Equation (3) needs to be improved. After the improvement, if *z* is the vertical vibration acceleration caused by the abnormal road surface, it can be described as Equation (6).

(6) $$\left|\left(z-\mu \right)\right|>\left(\frac{v}{T_{V}}\right)*T_{G}*\sigma$$

In addition, when the vehicle passes an abnormal road surface, the vertical acceleration of the vehicle is large. Therefore, if *z* is the acceleration caused by passing through the abnormal road, *z* will satisfy the following Equation (7).

(7) $$z>\left(\frac{v}{T_{V}}\right)T_{Z}$$

Similar to the Gaussian background model, the improved Gaussian background model will also update the model parameters. When the acceleration *z* does not satisfy Equations (6) and (7), the data is considered as background data and will be used to update the background parameters. The parameter update formula is shown in Equation (5).

Where *v* is the current vehicle speed, and T_V_ is the speed threshold. Vehicle suspension parameters are different for different types of vehicles. The vibration response of the vehicles body will be significantly different when different types of vehicles pass through the same road surface. The original Gaussian background model has a fixed threshold *T_Z_*, which is obviously not applicable to different types of vehicles. Therefore, this paper proposes designing a fuzzy logic controller to optimize the Gaussian background model.

Due to the complexity of the vehicle system, it is difficult to establish accurate mathematical models to describe the relationship between different vehicles and abnormal road surfaces. Fuzzy logic control is based on artificial experience and does not require an accurate mathematical model of the controlled object. Using fuzzy logic, the basic idea of optimizing the abnormal road surface recognition algorithm is: Firstly, find the fuzzy relationship between the road surface recognition algorithm parameter *T_Z_* and the vehicle suspension parameters. Second, in the process of road surface recognition, according to the difference of *K_S_* and *C_S_* parameters of different vehicles, fuzzy logic is used to modify *T_Z_*, so that the abnormal road surface recognition algorithm can adapt to different types of vehicles.

In this section, to make the abnormal road recognition algorithm applicable to different types of vehicles, a fuzzy logic inference machine is used to calculate an appropriate *T_Z_*. The input quantities of the fuzzy logic inference machine are defined as the vehicle suspension stiffness *K_S_* and damping *C_S_*. The basic domain of *K_S_* is [0,200], the basic domain of *C_S_* is [0,4], and the basic domain of *T_Z_* is [0.5,1].

**Figure 8:**
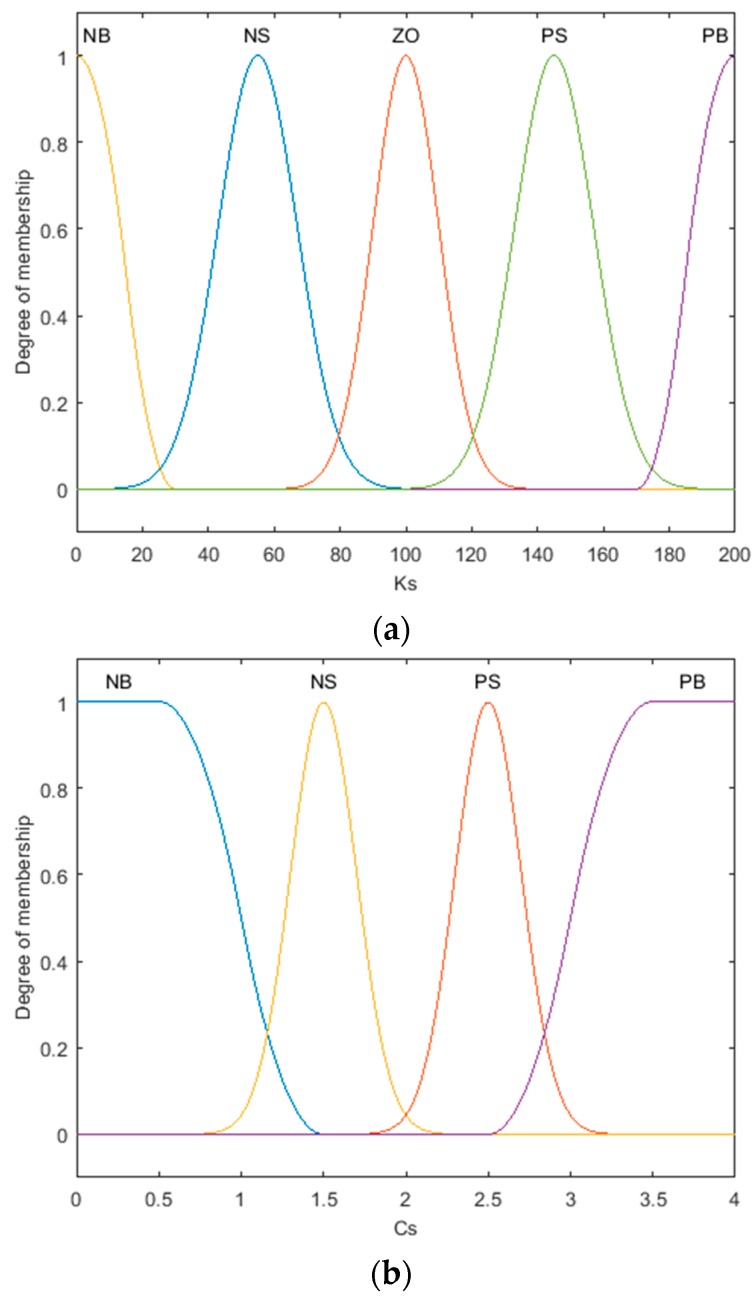

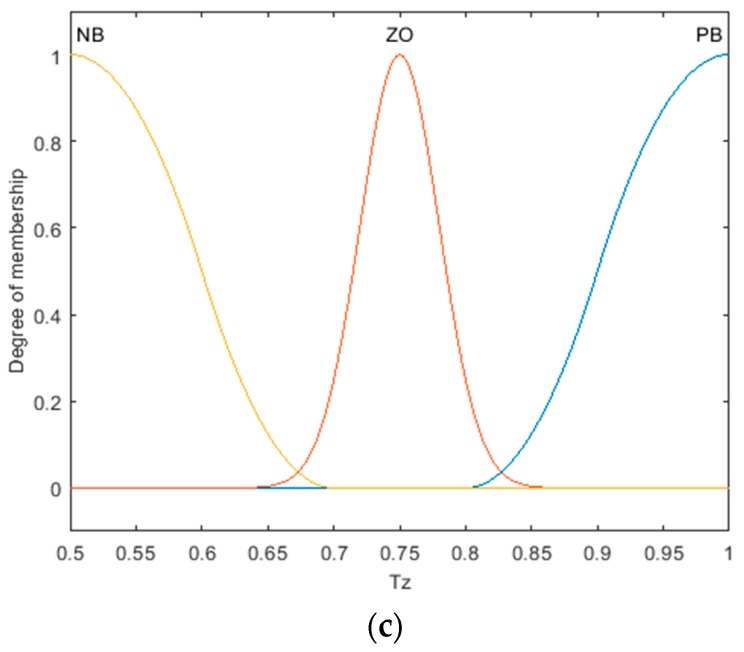
Membership function. (**a**) Membership function of input 1 (*K_S_*). (**b**) Membership function of input 2 (*C_S_*). (**c**) Membership function of the output (*T_Z_*).

As shown in Figure 8, the membership function is gaussmf type. The input 1 (*K_S_*) is qualitative into five sets, denoted as NB, NS, ZO, PS, and PB. The input 2 (*C_S_*) is qualitative into four sets, denoted as NB, NS, PS, and PB. The output (*T_Z_*) is qualitative into three sets, denoted as NB, ZO, and PB, where NB is negative big, and PB is positive big.

**Figure 9:**
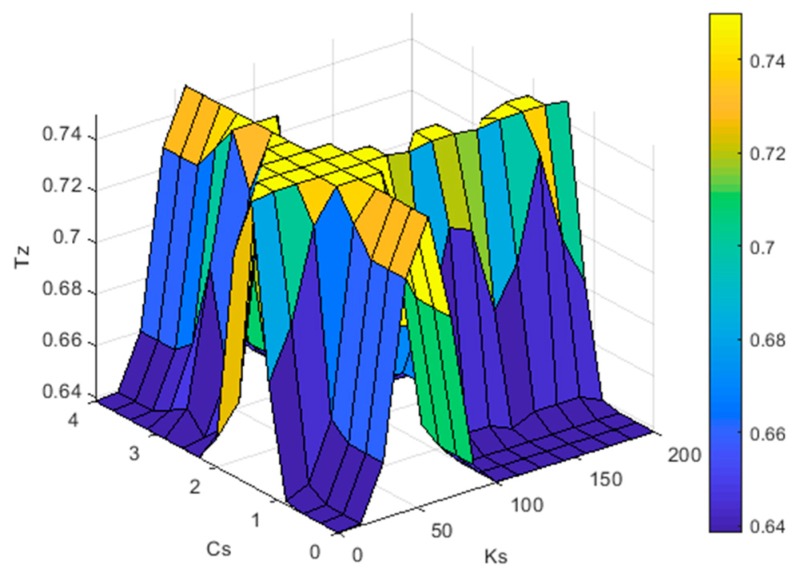
The surface of fuzzy logic controller’s output.

The establishment of the rule bases of the fuzzy logic inference machine is based on the experiments and CarSim simulation. The rule bases of the fuzzy logic inference machine are shown in Table 1, and Figure 9 shows the interface of the output.

The improved Gaussian background model is described in Algorithm 1.

**Algorithm 1:** Abnormal road surface recognition method **Input:**
*z*, the Z-axis acceleration; *v*, the vehicle speed; *K_S_*, the spring stiffness; *C_S_*, the damping coefficient.  **Output:*** event_z*, Acceleration due to abnormal road surface.  1. **Algorithm begin:** * 2. μ* = 0   *% μ* is a mathematical expectation  3. *σ* = 0   *% σ* is a standard deviation  4. T_G_ = 2  % T_G_ is the Gaussian matching threshold  5. T_V_ = 20  % T_V_ is the speed threshold  6. if (*v* > T_V_)  7.  *z_match* ←abs(*z* − *μ*)/*σ*
  8. % calculating thresholds *T_Z_* using fuzzy logic inference machines  9.  *T_Z_*← fuzzy_control (*K_S_*, *C_S_*)  10.  if (*z_match* > T_G_*v/T_V_) && (abs(*z*) > (T_Z_* *v*/T_V_))  11.   *event_z ← z*  12.  else    % update *μ* and *σ*, as described in Equation (5)  13.   *μ* ← (1 − α) * *μ* + α*z  14.   *σ* ← SQRT ((1 − α) * *σ*^2 + α * (*z* − *μ*)^2)  % SQRT means calculate square root  15.  end if  16. end if  17. return *event_z*  18. **Algorithm end**

## 5. kNN Algorithm Abnormal Road Surface Classification

The kNN classifier is a sample-based machine learning algorithm. Due to the simple and effective characteristics of kNN, kNN has been widely used in engineering applications [29]. First, the abnormal road surface acceleration signal extracted by the abnormal road surface recognition algorithm is used to identify the k nearest neighbor values in the training data set. Then, the abnormal pavement labels corresponding to these nearest neighbor values are counted, and the number of neighboring samples belonging to each possible type is calculated. The most common type of pavement that belongs to most of the k nearest neighbors is the type of pavement being measured.

### 5.1. Training and Testing Sample Data Sets

Both the training samples and the test samples are collected by the acceleration sensor of a smartphone. In this study, multiple sets of acceleration data caused by abnormal road surfaces are collected as training data sets. In addition, in order to maintain the independence between training data and test samples, the training samples and test samples are collected on two different roads. The number of training and test samples obtained in this paper is shown in Table 2.

**Figure 10:**
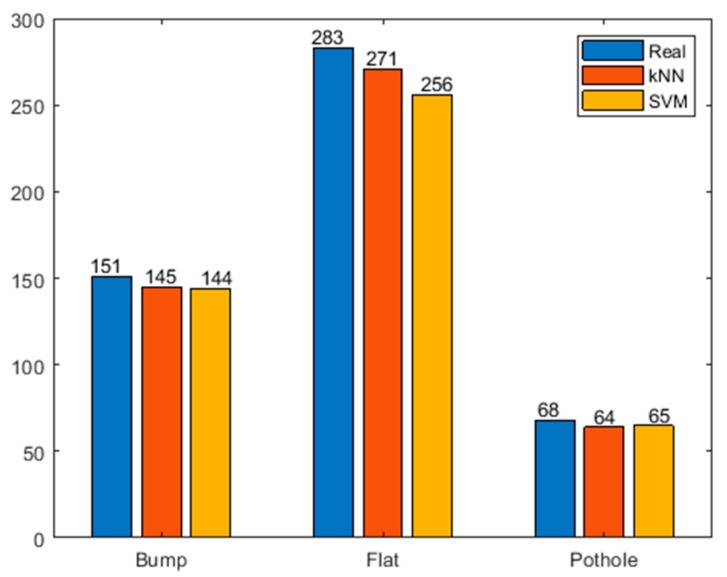
The performance of kNN and SVM.

From the perspective of the classification process, kNN most directly establishes a relationship between training samples and test samples, which can effectively avoid the negative impact caused by the improper selection of category features. Another widely used classification algorithm support vector machine (SVM) is utilized to compare with kNN on classifying the road surface. The same training and testing samples are used and the results are shown in Figure 10. The results show that kNN has an advantage over SVM in the classification of abnormal roads.

### 5.2. Classification Algorithms and Tuning Parameters

**Figure 11:**
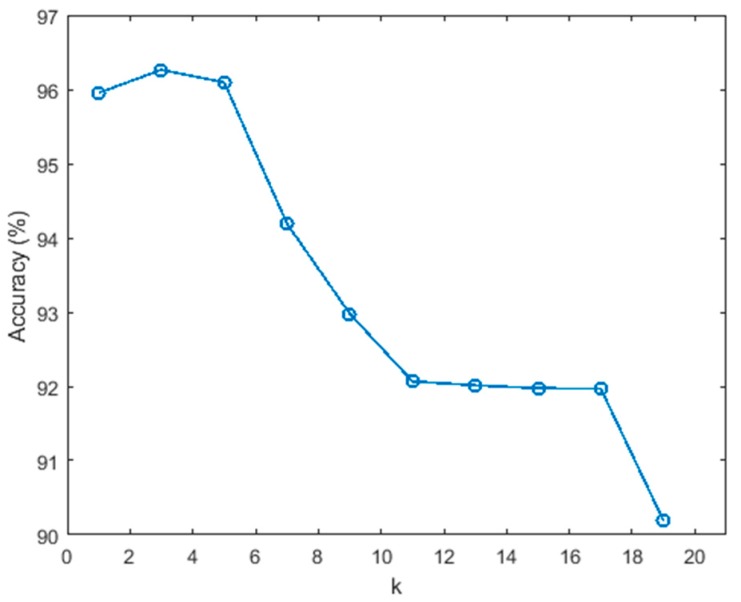
The relationship between classification accuracy (y-axis) and k value (x-axis).

The kNN classification algorithm classifies objects based on the attributes of k neighbors. The value of k is a key parameter of the kNN algorithm. This paper tests the classification effect when k takes different values in the range of 1–19 (odd numbers). Figure 11 shows the classification accuracy when k takes different values. It can be seen from the figure that as the value of k increases, the classification accuracy decreases, and the effect is best when k = 3. Therefore, k is set as 3 in this work.

## 6. Test and Analysis

### 6.1. Test Conditions

**Figure 12:**
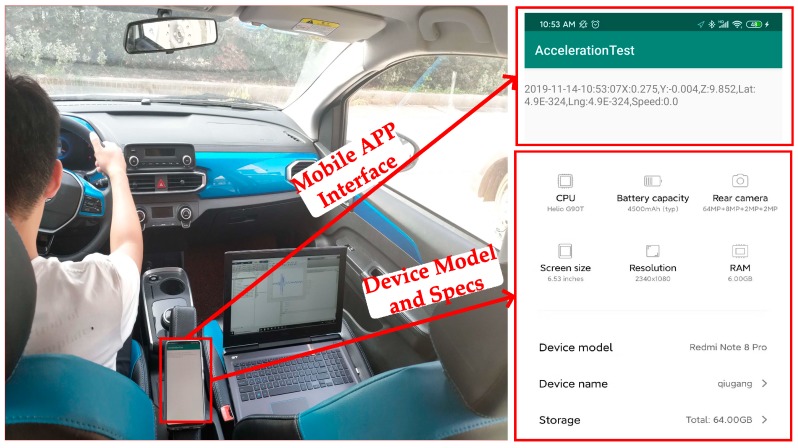
Smartphone installation location.

To validate the performance of the proposed algorithm, an A-class vehicle (Cavalier) and SUV (Qoros 5) are used to perform the test. An app working on a smartphone (Redmi Note 8 Pro) is developed to collect the acceleration, speed, and position. The sampling frequency is 400 Hz. According to the sampling theorem, if the frequency information of a signal is to be saved, the sampling frequency must be twice as much as the frequency of the measured object. If the amplitude information of a signal is to be saved, the sampling frequency is preferably ten times as much as the frequency of the measured object. The sampling frequency of vehicle speed and position is 1 Hz. This is because the GPS module data update frequency in the smartphone used in the experiment is 1 Hz. The smartphone is fixed on the handrail of the driver’s seat during the test, as shown in Figure 12.

**Figure 13:**
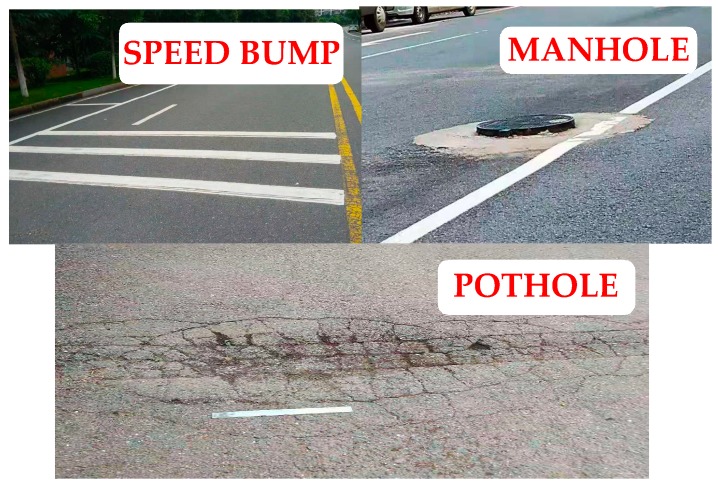
Abnormal road surfaces on the test road.

The vehicle travels at a different speed on an abnormal road and a flat road and performs multiple tests. There are many abnormal road surfaces on the test road, as shown in Figure 13. The actual measurement shows that the area of the pothole on the road is about 1 square meter and the maximum depression depth is 30 mm. The diameter of the raised manhole cover is 700 mm, and the height is 50 mm. The width of the speed bump is 350 mm, and the height is about 30 mm.

### 6.2. Vehicle Dynamic Response under Different Road Excitations

**Figure 14:**
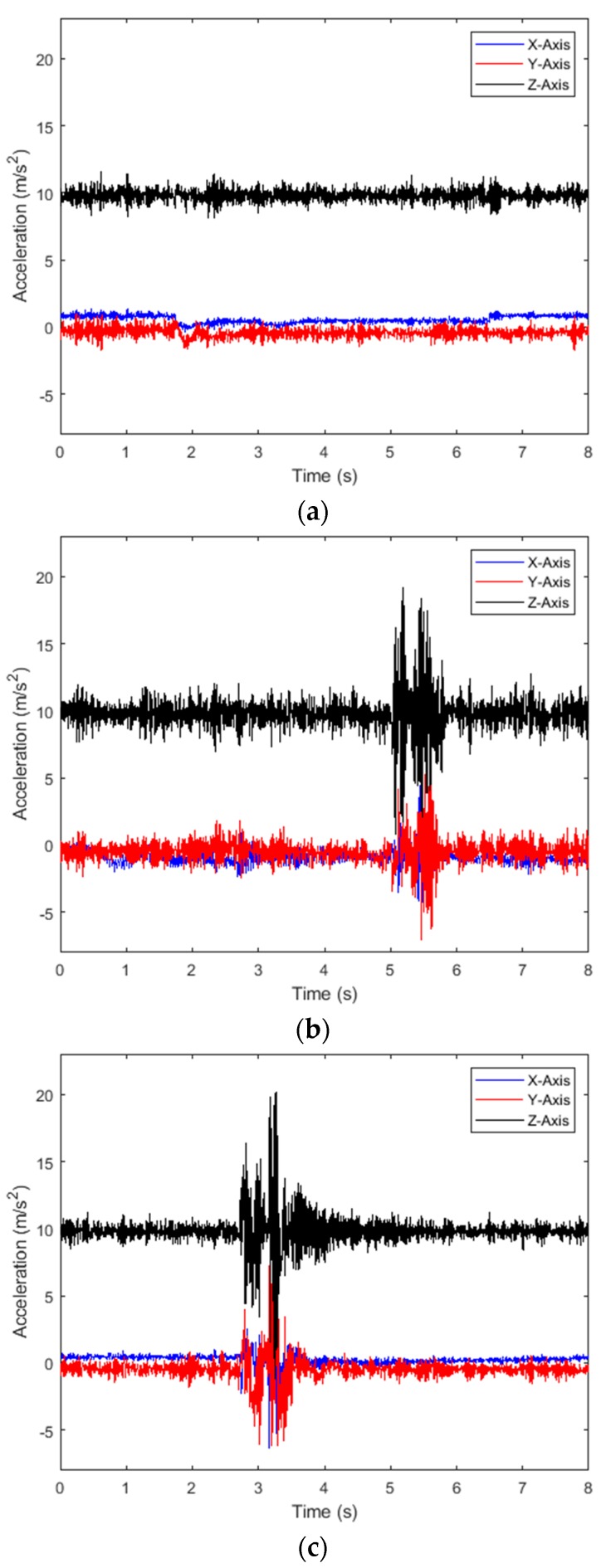
Vehicle vibration acceleration signal. (**a**) Flat road surface; (**b**) pothole road surface; (**c**) bump road surface.

When the vehicle is travelling on a flat road surface, its vibration acceleration is relatively stable. When the vehicle passes through an abnormal road surface such as a road pit or a road surface bump, the acceleration of the vehicle in the vertical direction changes significantly. Figure 14 shows the vibration acceleration of the vehicle when the vehicle passes through different roads at a speed of 30 km per hour. Figure 14a shows the vehicle traveling on a flat road surface. In Figure 14b, the vehicle is excited by the pothole road surface. In Figure 14c, the vehicle is excited by the bump road surface. Under the influence of gravity acceleration, the Z-axis acceleration fluctuates around 9.8.

**Figure 15:**
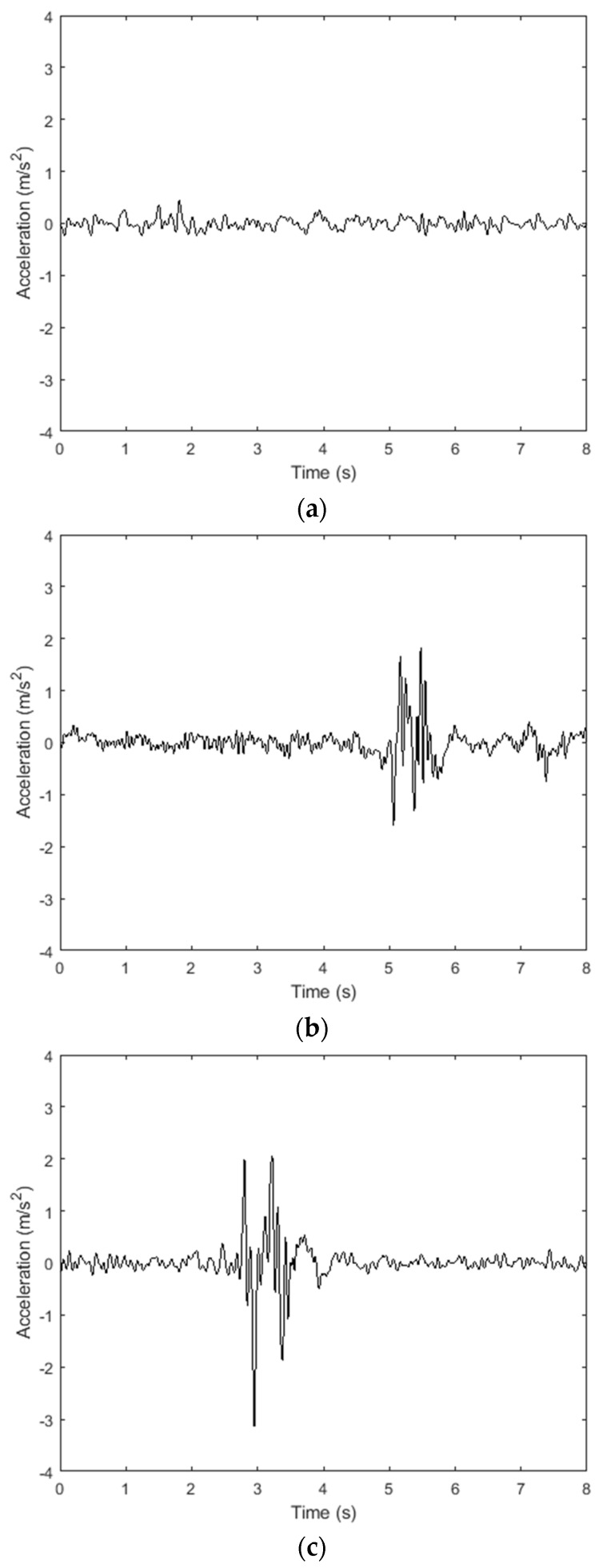
Z-axis vibration acceleration after filtering. (**a**) Flat road surface; (**b**) pothole road surface; (**c**) bump road surface.

After the original acceleration data is processed by the Butterworth filter, the filtered Z-axis vibration acceleration shown in Figure 15 is obtained. It can be clearly observed that the valid data collected by the smartphone is retained, and the through component and the high-frequency noise are eliminated.

### 6.3. Test Results and Analysis

**Figure 16:**
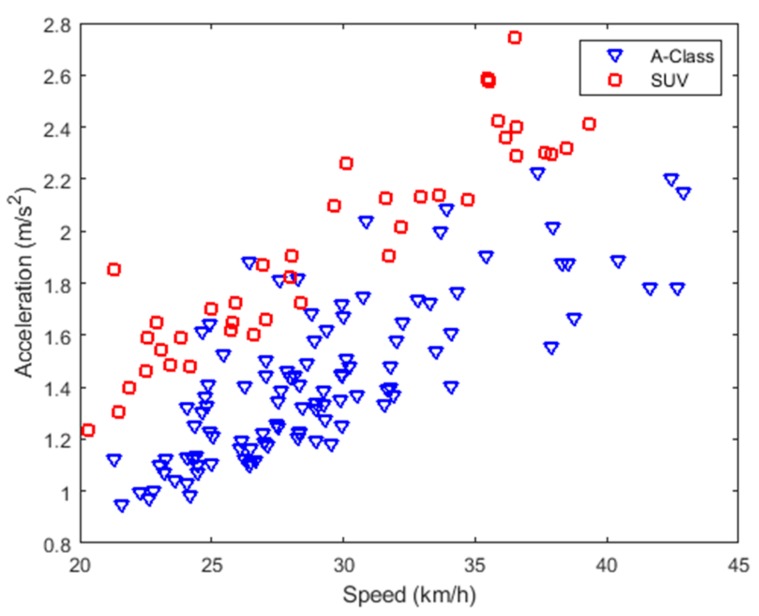
Vehicle speed and body vibration acceleration.

Figure 16 depicts the vertical acceleration values of the vehicle body when the experimental vehicle passes through the same abnormal road surface at different vehicle speeds. The blue triangle in Figure 16 represents the experimental data of the A-class vehicle, and the red rectangle represents the experimental data of the SUV.

It can be observed from Figure 16 that as the vehicle speed increases, the vertical vibration acceleration of the vehicle body also increases. In addition, when the different types of vehicles pass the same abnormal road surface, the vertical vibration acceleration of the vehicle body also has a significant difference.

Comparing the field measurement results with the algorithm identification results, as shown in Table 3, the results show that the proposed method can effectively identify the road surface potholes and bumps. In the 68 sets of road surface pothole data, 64 groups are successfully identified and classified, and the accuracy rate is 94.12%. Among the 151 sets of road surface bumps data, 145 groups are successfully identified and classified, and the accuracy rate is 96.03%. Through onsite investigation of the reported road surface, it is found that the abnormal road surface size of the false alarm is small, or the multiple abnormal road surfaces are close to each other, which leads to erroneous recognition results.

## 7. Conclusions

In this paper, a method for abnormal road surface recognition using a smartphone acceleration sensor is proposed. The Gaussian background model is optimized by a fuzzy logic inference machine so that the road surface recognition algorithm can be applied to different types of vehicles. Vehicle acceleration, speed, and position data are collected by the built-in acceleration sensor and global positioning navigation system of the smartphone. The vibration acceleration caused by the abnormal road surface is extracted using the improved Gaussian background model and the Z-axis acceleration threshold condition. An adaptive adjustment mechanism based on vehicle speed is proposed to improve the recognition accuracy. The classification of the abnormal road surface is realized by utilizing the kNN classification algorithm. Multiple sets of samples are used to test the abnormal road surface identification method. Comparing the algorithm identification results with the artificial site survey results, it is found that the proposed method can effectively identify and classify abnormal road surfaces such as potholes and bumps.

It is worth noting that with the increase of the total mileage of the road, the intelligent transportation system will be more and more widely used in the transportation industry. In this paper, only the two main types of the abnormal road surface are identified. The next step would be studying the evaluation and identification methods of the degree and size of abnormal road surface damage.

## Figures and Tables

**Table 1:** Rule bases of *T_Z_*.

	*K_S_*	NB	NS	ZO	PS	PB
*C_S_*						
NB		ZO	NB	NB	NB	NB
NS		NB	PB	NB	NB	NB
PS		ZO	NB	NB	ZO	NB
PB		ZO	NB	ZO	ZO	ZO

**Table 2:** Number of training and testing sample.

Road Surface	Training	Testing
Bump	118	151
Flat	174	283
Pothole	103	68

**Table 3:** Abnormal road surface identification result.

Road Surface	Field Measurement	Algorithm Identification	Accuracy Rate
Pothole	151	145	96.03%
Bump	68	64	94.12%
